# Biopolymeric Nanocomposites for CO_2_ Capture

**DOI:** 10.3390/polym16081063

**Published:** 2024-04-11

**Authors:** Rosalia Maria Cigala, Giovanna De Luca, Ileana Ielo, Francesco Crea

**Affiliations:** Dipartimento di Scienze Chimiche, Biologiche, Farmaceutiche e Ambientali, Università degli Studi di Messina, V.le F. Stagno d’Alcontres 31, 98166 Messina, Italy; rosaliamaria.cigala@unime.it (R.M.C.); giovanna.deluca@unime.it (G.D.L.); francesco.crea@unime.it (F.C.)

**Keywords:** carbon dioxide, CO_2_ capture, adsorption capacity, biopolymer, nanocomposite materials

## Abstract

Carbon dioxide (CO_2_) impacts the greenhouse effect significantly and results in global warming, prompting urgent attention to climate change concerns. In response, CO_2_ capture has emerged as a crucial process to capture carbon produced in industrial and power processes before its release into the atmosphere. The main aim of CO_2_ capture is to mitigate the emissions of greenhouse gas and reduce the anthropogenic impact on climate change. Biopolymer nanocomposites offer a promising avenue for CO_2_ capture due to their renewable nature. These composites consist of biopolymers derived from biological sources and nanofillers like nanoparticles and nanotubes, enhancing the properties of the composite. Various biopolymers like chitosan, cellulose, carrageenan, and others, possessing unique functional groups, can interact with CO_2_ molecules. Nanofillers are incorporated to improve mechanical, thermal, and sorption properties, with materials such as graphene, carbon nanotubes, and metallic nanoparticles enhancing surface area and porosity. The CO_2_ capture mechanism within biopolymer nanocomposites involves physical absorption, chemisorption, and physisorption, driven by functional groups like amino and hydroxyl groups in the biopolymer matrix. The integration of nanofillers further boosts CO_2_ adsorption capacity by increasing surface area and porosity. Numerous advanced materials, including biopolymeric derivatives like cellulose, alginate, and chitosan, are developed for CO_2_ capture technology, offering accessibility and cost-effectiveness. This semi-systematic literature review focuses on recent studies involving biopolymer-based materials for CO_2_ capture, providing an overview of composite materials enriched with nanomaterials, specifically based on cellulose, alginate, chitosan, and carrageenan; the choice of these biopolymers is dictated by the lack of a literature perspective focused on a currently relevant topic such as these biorenewable resources in the framework of carbon capture. The production and efficacy of biopolymer-based adsorbents and membranes are examined, shedding light on potential trends in global CO_2_ capture technology enhancement.

## 1. Introduction

Carbon dioxide (CO_2_) is a gas which plays a crucial role in the greenhouse effect, which, when enhanced, has led to global warming and climate change. For this reason, CO_2_ capture, also known as carbon capture, describes the process of capturing this gas, among the other emissions produced by various industrial processes or power generation facilities before they are released into the atmosphere. The primary objective of CO_2_ capture is to reduce greenhouse gas emissions, mitigating the impact of human activities on climate change. Biopolymer nanocomposites have emerged as promising materials for CO_2_ capture due to their renewable and sustainable nature [1]. These materials are composed of biopolymers (naturally occurring polymers derived from biological sources) and nanofillers, such as nanoparticles, nanotubes, or nanosheets, which enhance the properties and performance of the biopolymer matrix. Various biopolymers have been investigated, including chitosan, cellulose, carrageenin, starch, alginate, and proteins like soy and zein. These biopolymers possess unique functional groups that enable them to interact with CO_2_ molecules.

In some research, nanofillers are incorporated into the biopolymer matrix to improve its optical, mechanical, thermal, and sorption properties. Commonly used nanofillers include graphene, carbon nanotubes, metallic nanoparticles, metal–organic frameworks (MOFs), and zeolites [2,3,4]. The CO_2_ capture mechanism in biopolymer nanocomposites primarily involves chemisorption and physisorption. Functional groups in the biopolymer matrix, such as amino and hydroxyl groups, facilitate CO_2_ adsorption through weak interactions [5,6]. The addition of nanofillers increases the surface area and porosity of the biopolymer nanocomposites, leading to enhanced CO_2_ capture volume. Many advanced materials have been developed for absorption, adsorption, and membrane separation, such as biopolymeric derivatives, which are attractive components for CO_2_ capture technology. Biopolymers such as cellulose, alginate, chitosan, carrageenan, and their derivatives are accessible and inexpensive. Therefore, this review is focused on the more recent studies on biopolymer-based materials employed for CO_2_ capture strategies; it offers an overview of composite materials based on cellulose, alginate, chitosan, and carrageenan biopolymers enriched with nanomaterials and explores their future potential.

The main contribution of polymer nanocomposites for CO_2_ capture lies in their potential to address two critical challenges facing industries and sustainability efforts: reducing greenhouse gas emissions and mitigating climate change. By incorporating nanomaterials into biopolymer matrices, these composites can effectively capture and adsorb CO_2_ from industrial flue gases and other emission sources. The use of polymer nanocomposites for CO_2_ capture can revolutionize various industrial processes, particularly in power plants, cement production, and other high-emission sectors. These materials can be deployed in carbon capture and storage systems to reduce CO_2_ emissions directly from exhaust streams before they are released into the atmosphere. Industrial actors can significantly reduce their carbon footprint and comply with increasingly stringent emissions regulations by integrating polymer nanocomposites into existing infrastructure. This helps mitigate climate change and develop a circular carbon economy where CO_2_ is treated as a value-added resource rather than a waste product.

This semi-systematic literature review concentrates on recent research concerning biopolymer-based sorbent materials utilized for CO_2_ capture. It offers insights into composite materials enhanced with nanomaterials, with a specific emphasis on cellulose, alginate, chitosan, and carrageenan. These are among the most relevant marine biopolymers that are already profitably employed in food packaging and drug delivery, to name only a few applications [7,8]. As we hope to demonstrate in this review, the biocompatibility and sustainable nature of the biopolymers mentioned above provide them with a pivotal perspective role in CO_2_ capture, storage, and reuse strategies.

The preparation and efficiency of biopolymer-based adsorbent and membrane materials are examined to investigate the possible improvement of CO_2_ capture technologies globally.

## 2. CO_2_ Capture Mechanism

The CO_2_ capture mechanism primarily refers to removing carbon dioxide from the atmosphere or other environments. This phenomenon occurs through various natural and artificial processes that play a crucial role in the global carbon cycle and, consequently, in influencing the Earth’s climate. Carbon capture and storage (CCS) or sequestration are among the most studied technologies for capturing CO_2_ emissions from power plants, industrial facilities, and other sources. CO_2_ is captured, transported, and then trapped in underground geological formations, like exhausted oil fields or saline aquifers, to prevent it from being released back into the atmosphere. These technologies are considered an essential component of efforts to mitigate greenhouse gas emissions and fight global warming. 

**Figure 1:**
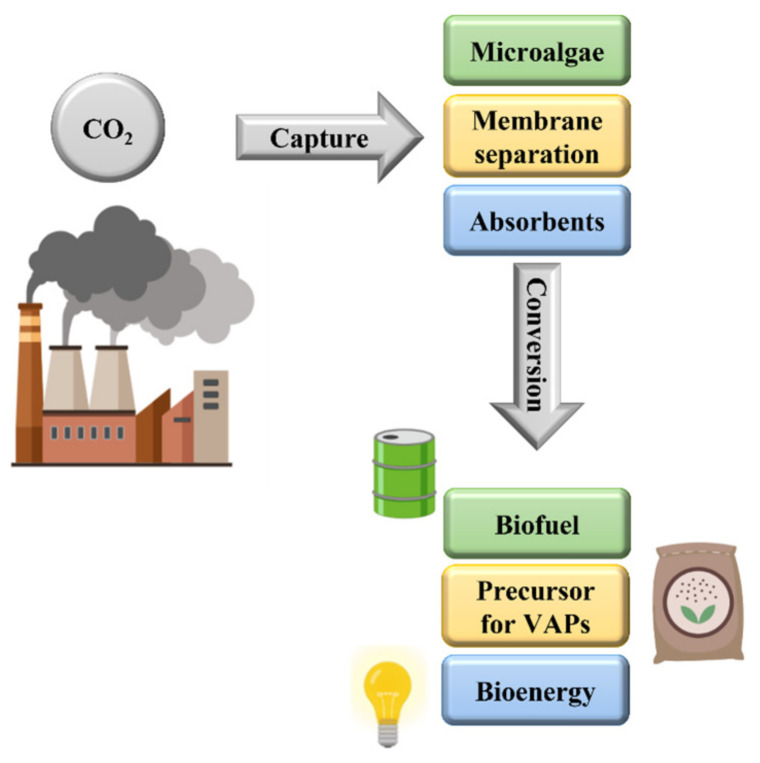
Schematic illustration of CO_2_ capture and conversion methodology and utilization [11].

The present CO_2_ storage approach is highly energy-intensive and has elevated operation costs. The reduction in CO_2_ levels, according to conventional procedures, occurs through direct atmospheric capture, followed by separation and storage [9]. The direct capture of CO_2_ from the air generates a stream of pure CO_2_ as the primary income for numerous industries [10]. However, the captured CO_2_ may be upcycled and transformed into more profitable value-added products (VAPs) [1]. The existing industrial methods to produce VAPs include chemical, biological, photochemical, and electrochemical transformations. Figure 1 shows a cartoon representation of the main ways of converting and using CO_2_.

Three methods are used for CO_2_ capture and storage [12]:

1. Post-combustion capture, where CO_2_ is captured after fuel combustion.
2. Pre-combustion capture, where CO_2_ is captured before it is released into the atmosphere.
3. Oxy-fuel combustion, which involves burning fossil fuels using oxygen (O_2_) and recycled flue gas as a substitute for air.

All of them are complex and expensive for industrial settings [1].

Microalgae and their derivatives are excellent raw materials for CO_2_ emission reduction, and their absorption capacity is 10–50 times higher than terrestrial microalgae [13]. This process naturally occurs in aquatic environments where microalgae are present. However, in the context of carbon capture technologies, researchers are exploring ways to enhance this process for industrial-scale CO_2_ capture [13]. One approach is to cultivate microalgae in controlled environments such as photobioreactors, where CO_2_-rich flue gases from industrial processes can be bubbled through the algae culture [11]. The microalgae absorb the CO_2_ and utilize it for photosynthesis, thereby capturing carbon from the gas stream. Among others, factors such as light intensity, temperature, nutrient availability, and CO_2_ concentration can influence the efficiency of CO_2_ capture by microalgae. Many researchers have explored the advantages of employing microorganisms to capture CO_2_ from the environment, simultaneously generating biodiesel [14,15,16,17]. Besides the active role of microalgae in CO_2_ capture, we can also point out their role as source of derivative materials that can find application in this same field. Carrageenan, for example, is extracted from a certain species of red seaweeds, which are types of marine algae. It has been studied for its potential application in capturing carbon dioxide, limiting its release into the air, and storing or using it in a controlled manner. This approach, presently employed only on a laboratory scale, may become a sustainable alternative in CO_2_ storage methods as extensive research on storage and recovery may contribute to the success of the model in the coming years [18]. 

Membrane separation is one of the promising technologies for CO_2_ capture because it offers the potential for low energy consumption and reduced environmental impact compared to traditional absorption-based methods. Here, porous membranes selectively separate CO_2_ from a gas mixture, such as flue gas from industrial processes, thanks to the chemical and physical interactions between the various gases and the biomaterials constituting the membranes [19]. The membrane allows CO_2_ to pass through while blocking other gases, either by means of size discrimination or taking advantage of different chemical interactions. Different types of membranes, such as polymeric, mixed matrix, and ceramic ones, have been developed and studied for CO_2_ capture applications. The efficiency of membrane separation depends on factors such as the nature of membrane materials, pore size, operating conditions (pressure, temperature), and gas composition [20]. In this framework, biopolymeric nanocomposites are materials with excellent potential as green membrane constituents to be applied in environmentally sustainable greenhouse gas absorption technologies.

Thus, several polymer membranes such as organic, polymer materials, or inorganic carbons, ceramics, [6,21], zeolites [22], metal–organic frameworks (MOFs) [23], and silica [24,25] have been largely studied [26,27,28]. Compared with other separation methods, such as the nanoparticle absorption and adsorption process, membrane separation has the advantage of regeneration demanding less energy and can be combined with complementary technologies [29]. However, this approach shows specific difficulties, as in the case of exhaust gases, where CO_2_ is emitted at low pressure and concentration, both being unfavorable conditions that cause a reduction in the efficiency of CO_2_ capture [29,30] In fact, these selective semi-permeable polymer-based membranes can simply capture CO_2_ from air flow [31,32]. 

Another eco-sustainable and low-cost technology for capturing CO_2_ consists of carbon-based materials produced from renewable sources, which show quick adsorption/desorption kinetics [33]. The production process for these adsorbents, in terms of energy consumption, labor costs, equipment maintenance, and process efficiency, influences production costs. For example, the synthesis of MOFs typically involves energy-intensive processes such as solvothermal reactions. In addition, adsorbents used for CO_2_ capture typically need to be regenerated periodically to remove the captured CO_2_ and restore their adsorption capacity. Regeneration processes often involve heating the adsorbent to release the captured CO_2_, which requires energy input. The associated costs depend on factors such as the energy efficiency of the regeneration process, the temperature and pressure conditions required, and the frequency of regeneration cycles. 

Materials such as biomass, polymeric sorbents, and activated charcoal capture CO_2_ through adsorption and desorption processes. Their adsorption capacity is mainly related to the chemical structure, surface area, pore size and morphology, and surface reactivity. [34]. Drawbacks are present; e.g., activated charcoal is unsuitable for the selective adsorption of gases due to its highly heterogeneous porous structure [35,36]. However, revalorizing biomass to obtain materials with high added value for CO_2_ capture is an excellent approach to implementing environmental sustainability policies. The two principal procedures for improving the CO_2_ capture and separation capability of the activated carbon surface are to graft nitrogen-containing functional groups and metal oxides, such as MgO and CaO [37]. The low affinity of raw activated charcoal for CO_2_ is improved thanks to doping with amine nitrogen, which attacks the electrophilic carbon of CO_2_ as a nucleophile, binding it covalently [38].

### Enhancing CO_2_ Capture Capacity

Absorption, adsorption, and membrane separation are classic approaches for CO_2_ capture that are deeply enhanced by any improvement in the nanocomposite constituents. 

Biphasic solvents, for example, convert into two phases, liquid–liquid or liquid–solid, resulting in absorption or temperature modification, and they facilitate the chemical absorption of amines and ammonia compounds [39,40]. Biphasic solvents for CO_2_ capture include, namely, amine/alcohol mixtures and ionic liquids such as imidazolium salts, which have excellent thermal stability [41]. In the latter case, the anionic moiety promotes CO_2_ absorption, whereas the nature of the cations affects CO_2_ solubility. However, once CO_2_ has been absorbed, much energy is required for the material to be reused and to regenerate the absorbent substance. For this reason, another valid approach is adsorption.

**Figure 2:**
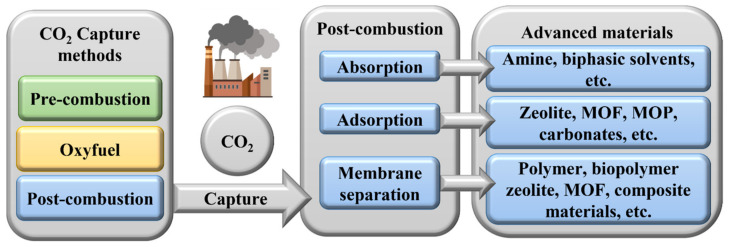
Advanced materials for CO_2_ capture post-combustion methods.

Adsorption implies the physical or chemical trapping of CO_2_ into the pores of materials such as carbonaceous particles, zeolites, MOFs, microporous organic polymers (MOPs), and amine-modified particles, as represented in Figure 2. 

If the igneous carbon nanostructured materials, such as those found in biochar and carbonized biomass, are the cheapest alternatives in an adsorbent variety [42], 0-D to 2D carbon allotropes, i.e., fullerenes, carbon nanotubes, and graphene, exhibit an improved surface area and enhanced surface chemistry for CO_2_ capture [43]. 

Zeolites and their derivatives are crystalline and microporous aluminosilicates with well-defined pore sizes presenting high CO_2_ selectivity and adsorption capacity [3]. MOFs are crystalline materials with microporous structures with tunable porosity, which can be tailored to host CO_2_ molecules (0.33 nm) specifically. CO_2_ capture is further improved by means of a polar functionalization of the pores’ surface, such as that provided by grafting –OH and –NH_2_ moieties [2].

MOPs are characterized by a porous structure with pores having dimensions <2 nm and are suitable for CO_2_ capture [44]. 

**Figure 3:**
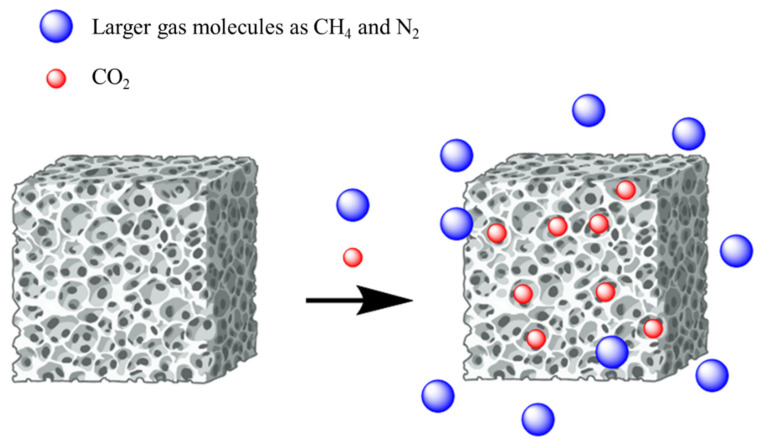
Gas adsorption on microporous biopolymer.

Chemical modification is indeed a feasible approach for designing porous materials with improved CO_2_ adsorption capabilities, both to obtain a better selectivity for this gas (Figure 3) and a higher adsorption capacity.

In this framework, biopolymers including cellulose, alginate, chitosan, and κ-carrageenan show great potential thanks to their rich chemistry, providing derivatives with excellent physical properties by design, which can be employed in the fabrication of CO_2_-selective and low-cost membranes. Carbon dioxide separation from dinitrogen and methane is made possible by using biopolymeric membranes with specific molecular porosity. Microporous particles may also be incorporated into the membranes by functionalization and crosslinking reactions to create nano- (0.7–2 nm) and ultrananopores (<0.7 nm) to improve CO_2_ permeability and selectivity [45].

## 3. Biopolymers for CO_2_ Capture

### 3.1. Cellulose

Cellulose is a ubiquitous biopolymer consisting of numerous D-glucose monomeric units linked via a β-1,4 glycosidic bond. It is the most abundant structural component in plants and the most abundant renewable organic polymer on our planet [46].

**Figure 4:**
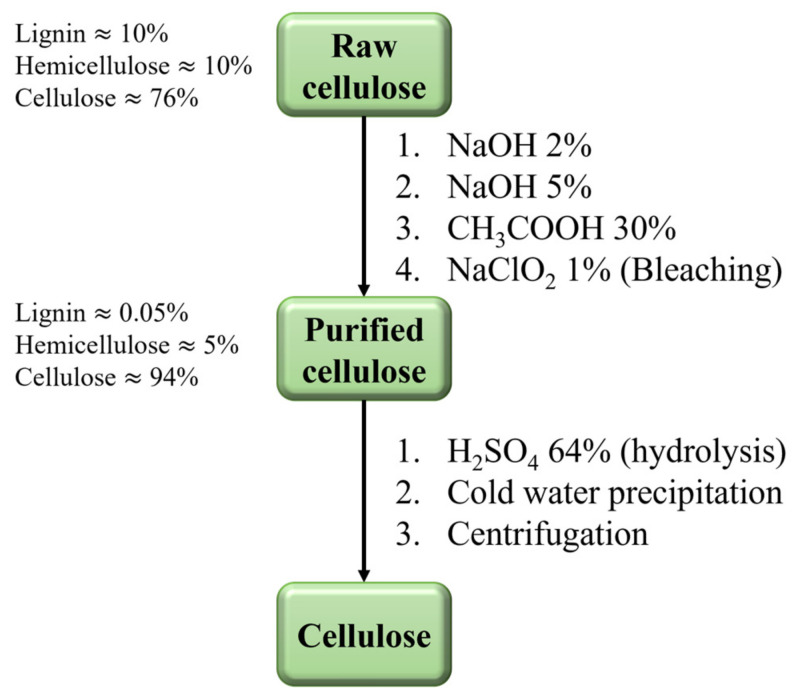
Flowchart of the cellulose synthesis process [49].

Employing biodegradable and non-toxic cellulose and its derivatives in CO_2_ capture methods is a sustainable choice due to the broad bioavailability of this polymeric matrix. Industrial cellulose synthesis typically involves processing cellulose-rich materials, such as wood pulp, cotton linter, or agricultural residues, subjected to purification, dissolution, and regeneration processes, as summarized in the flow diagram in Figure 4. Cellulose nanofibrils (CNFs) can be mechanically isolated from lignin and hemicellulose via high-pressure homogenization, grinding, ultrasonication, microfluidization, or wet-chemical methods [47]. Although the diameter of CNFs is usually <10 nm, their extension reaches the micrometer range. CNFs are generally used to consolidate the structure or to alter the viscosity of a nanocomposite, improving its mechanical performance [48].

Another cellulose derivative is nanocrystalline cellulose (CNC), obtained through enzymatic treatment or acid hydrolysis and having the same diameter as the CNFs but a length of <100 nm. CNCs are widely utilized to improve the strain at the breakdown of the composite [47]. 

Alongside these cellulose derivatives being biodegradable and biocompatible, they cause reduced interfacial adhesion and extra-hygroscopicity, which are detrimental to environmental applications. Chemical modification is, therefore, often necessary to adapt the properties of nanocellulose and make it suitable for producing adsorbent materials for CO_2_ capture [50]. 

Table 1 briefly describes recent research focusing on the employment of cellulosic materials as CO_2_ adsorbents.

The primary approaches involve the chemical modification of cellulose via the incorporation of inorganic nanoparticles. There are few cellulose-based absorbent materials that show a CO_2_ absorption capacity comparable to that of the aforementioned porous materials typically used for this purpose. These include CNF aerogels chemically modified with phthalimide (1,3-dihydro-1,3-dioxoisoindole), and silica/CNC composites functionalized with triethoxysilylpropylpropyl-3 -pentanyldinitrile-carbamate [34].

**Figure 5:**
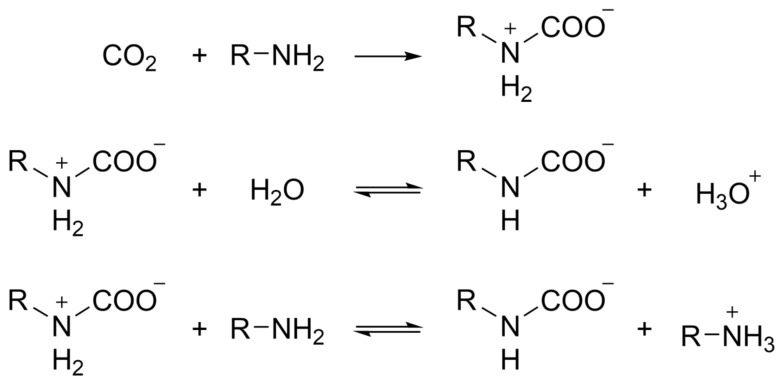
Schematic reaction between amine groups and CO_2_ and the influence of humidity content.

Similarly, the chemical functionalization of nanocellulose aerogels has been investigated to improve the selectivity toward CO_2_ in the development of CO_2_ adsorbents [33]. The nanocellulose –OH groups allow amino-silane surface modification to promote CO_2_ chemisorption. The modified CNF aerogel can adsorb 2.26 mmol/g of CO_2_ under dry conditions. The adsorption capacity of CO_2_ increases proportionally to the humidity up to 2.54 mmol/g, as the water molecules induce zwitterionic formation due to the interaction between CO_2_ and the primary amine of the silanes, resulting in the formation of the carbamate, as schematized in Figure 5.

Likewise, CNF aerogel was grafted by *N*-(2-aminoethyl)-3-aminopropylmethyldimethoxysilane in acetic acid to avoid the self-polymerization of the alkoxysilane while encouraging the reaction with –OH groups on the cellulose surface [55]. Chemisorption is thus facilitated under low-pressure conditions, making these cellulose-based materials suitable for removing CO_2_ from the exhausts.

With a different approach to the above-described studies, nanocellulose thin films were obtained from corn husks, oat hulls, and kraft pulp and modified using 3-aminopropyltriethoxysilane (APTES) and other silanes. Nanocellulose thin films improved by (3-trimethoxysilylpropyl)diethylenetriamine adsorbed the maximum amount of CO_2_ (2.11 mmol/g), with the adsorption capacity being influenced both by the high amine content but also by the lower surface area [67].

Likewise, the integration of triethoxysilylpropyl-3-pentanyldinitrilecarbamate-grafted silica nanoparticles within CNC [46] increased CO_2_ adsorption up to 5.54 mmol/g [53]. The effects induced by chemical functionalization are determining factors of CO_2_ adsorption, which decreased with increasing silica content, notwithstanding the growth in surface area; therefore, the role exerted by silica NPs was merely to improve gas selectivity.

Cellulose acetate membranes with a polydimethylsiloxane coating can efficiently separate CO_2_ from methane (CO_2_/CH_4_ selectivity: 43.8) [68]. Conversely, compared to uncoated cellulose acetate membranes, the coated ones exhibited better permeability but reduced selectivity when the CO_2_/N_2_ ratio was considered.

Cellulose acetate membranes were similarly combined with multi-walled carbon nanotubes functionalized with carboxyl groups, finding higher CO_2_/CH_4_ and CO_2_/N_2_ selectivity with 21.81 and 13.74 values, respectively. The employment of polyethylene glycol- and styrene butadiene-based rubbers as additives led to improved CO_2_/CH_4_ selectivity up to 53.98 and 43.91 ratios, respectively [67]. Some recent studies on CO_2_ separation and cellulosic membranes are summarized in Table 2, while Table 3 lists the main advantages and disadvantages of cellulose-based nanocomposites.

Functionalized nanocellulose composite materials have significant potential in CO_2_ adsorption, provided that their high surface-to-volume ratio is maintained after functionalization [86]. Further research should focus on desorption studies, particularly examining the material stability during regeneration at elevated temperatures. Furthermore, while inorganic nanoparticles could serve as a nanocellulose reinforcement, improvements to the surface area of chemically modified nanocomposites are needed to enhance the effectiveness of nanocellulose derivatives as adsorbents. In the end, regenerated cellulose nanocomposites deserve further investigation in CO_2_ adsorption applications [85].

Among cellulose-based membranes, regenerated cellulose mixed matrix membranes, polymeric membranes with nanocellulose incorporation, and nanocellulose-based membranes are selected as possible candidates for future study to scale up CO_2_ capture from flue gas, natural gas, and landfill gas.

### 3.2. Alginate and Chitosan

Alginate and chitosan are two distinct natural biopolymers with unique properties and applications. Both are derived from renewable resources and have found widespread use in various industries, including the food, pharmaceuticals, biotechnology, and biomedical fields [87,88].

**Figure 6:**
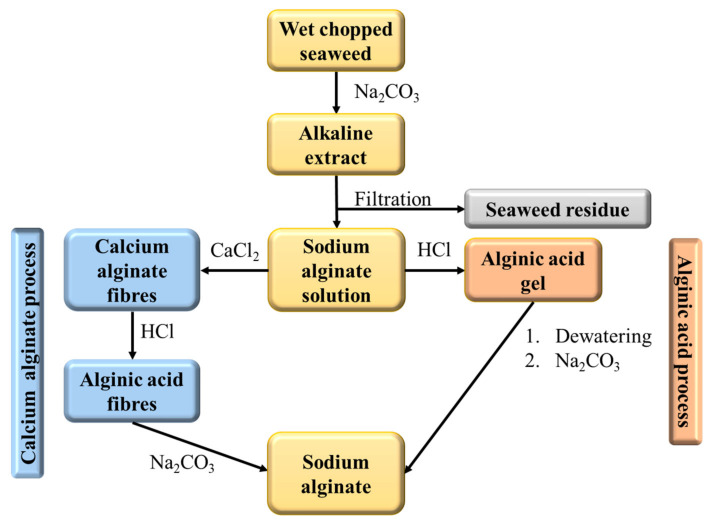
Flowchart of sodium alginate synthesis via two typical processes: calcium salt precipitation (blue boxes) or acidification (orange boxes).

Alginate is a polysaccharide obtained through extraction processes starting from brown marine algae and including the two typical approaches described in the flow diagram in Figure 6. It has garnered significant attention for the broadness of its applications, including biotechnology, biomedicine, drug delivery, and tissue engineering, for example, as an enzyme and protein immobilizer or as a template for fabricating nanocomposite materials [89]. Alginate is a hydrophilic, biodegradable, and non-toxic linear polysaccharide composed of two types of monomers: α-L-guluronic acid and β-D-mannuronic acid. These monomers can arrange in different sequences, leading to various types of alginates with different properties. One of the main reasons alginate is suitable for many applications lies in its chemical structure, which contains abundant carboxyl and hydroxyl groups. These functional groups display a pivotal role in immobilization and gelation processes, typical of this biopolymer, due to their chemical reactivity towards complementary moieties (e.g., amines) or calcium ions [90]. 

**Figure 7:**
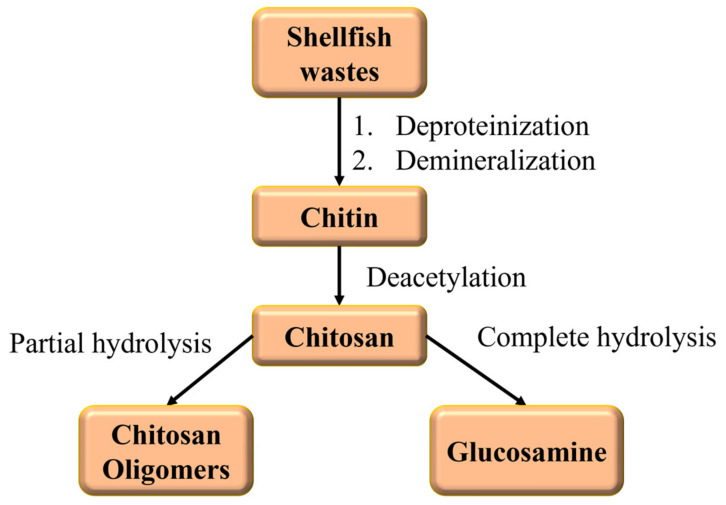
Flow diagram of chitosan synthesis and possible hydrolysis in the corresponding oligomers or monomers [93].

Chitosan is a biopolymer derived after the deacetylation of chitin, the latter being the second most abundant polysaccharide primarily found in fungi cells and arthropod exoskeletons [91,92]. It is a linear polysaccharide consisting of randomly distributed N-acetylated and deacetylated glucosamine units. Chitosan is now widely produced commercially from crab and shrimp-shell waste with several degrees of deacetylation and molecular weights; the generic production scheme is represented by the flow diagram in Figure 7. Chitosan is biodegradable, biocompatible, and has antimicrobial properties, being widely used in numerous applications, e.g., in drug delivery, tissue engineering, or as a flocculant in water treatment.

Alginate and chitosan are often used together to take advantage of their complementary electrostatic properties providing increased stability and durability to the resulting materials [94,95]. The combination of their unique properties makes alginate and chitosan valuable materials for a wide range of applications in different industries. 

The following paragraph aims to provide a comprehensive overview of the existing studies on CO_2_ capture using composites of alginate, chitosan, or their combination. The different preparation methods, the influence of varying composite compositions, and the mechanisms involved in CO_2_ capture will be analyzed.

Li et al. [96] discuss the synthesis of a composite material consisting of poly(vinyl alcohol) (PVA) and sodium alginate hydrogels for the preparation of robust and well-intergrown Zeolitic Imidazole Framework (ZIF) composite fiber membranes. The process involves introducing PVA–sodium alginate composite hydrogels to facilitate the synthesis of ZIF. These hydrogels act as nucleation sites leading to in situ defect-free MOF membrane fabrication. Furthermore, these membranes exhibit improved stiffness and durability due to rigid crystalline MOF layers. An additional advantage of this strategy is that it provides a versatile and general method for producing dense MOF membranes on various polymeric supports. These membranes demonstrated excellent performance in terms of a high H_2_ permeability equal to 9.66 × 10^−7^ mol m^−2^ s^−1^ Pa^−1^, as well as a notable H_2_/CO_2_ separation ratio up to 29.0 [96].

Another study described the preparation of a bio-degradable composite made of calcined egg-shell/sodium alginate beads as an adsorbent for CO_2_ capturing in a fixed-bed reactor. The beads were first synthesized and then functionalized using aqueous ammonia to introduce additional nitrogen-containing surface functional groups. The performance of the modified beads was examined under different experimental conditions, as different pressures (1 bar < *p* < 2.5 bar), temperatures (303 K < *t* < 323 K), flow rates (50 mL/min < flow rate < 90 mL/min), and inlet CO_2_ concentrations (20 to 45 vol%). The obtained results demonstrated that ammonia-impregnated beads had a higher CO_2_ adsorption capacity than non-impregnated ones. The highest CO_2_ adsorption capacity of 0.2380 mmol/g was achieved with the gel beads under the conditions of 1 bar and 303 K at a CO_2_ concentration of 45 vol%. The CO_2_ adsorption capacity was found to decrease with an increasing temperature, while it increased with higher inlet CO_2_ concentration, pressure, and flow rate. Regarding the CO_2_ adsorption/desorption process, the CO_2_ adsorption capacity of the gel spheres decreased in the second cycle but remained almost constant later on, suggesting an initial chemisorption step followed by subsequent physisorption cycles [97].

In a similar study, a biocomposite material made of amine-functionalized silica and alginate was investigated for its potential to efficiently capture CO_2_. The adsorption efficiency of the biocomposite was studied in a fixed-bed reactor, and breakthrough curves were plotted to assess the impact of the various experimental parameters. The optimum conditions for CO_2_ removal were determined at a temperature of 313 K, CO_2_ flow rate of 40 mL/min, and 5% humidity. Under these optimized conditions, the CO_2_ removal reached 7.865 mmol/g, showing that the silica content improved the adsorption processes [88]. 

Different studies focused on the immobilization of carbonic anhydrase (CA) and its impact on enhancing CO_2_ capture in post-combustion carbon capture processes. CA is an enzyme which catalyzes the conversion of CO_2_ to bicarbonate ions and immobilizes it in biopolymers, such as alginate and chitosan, using the cross-linking method. In particular, a recent study showed the immobilization process of CA in alginate which exhibited improved pH and thermal stabilities compared to its free form. Additionally, the immobilized CA showed increased resistance to chemical contaminations commonly present in exhausted gas scrubbing solutions. Batch-scale studies using the immobilized CA demonstrated that CO_2_ absorption rates were accelerated by the presence of the immobilized enzyme. The gas flow rate was a crucial factor influencing CO_2_ absorption when the CA load was low. However, as the gas flow increased, the enzyme load came to be the central factor modifying CO_2_ absorption [98].

Chitosan, similar to alginate, is combined with silica to obtain an adsorbent composite material for CO_2_ capture. The researchers coated chitosan on a high-surface-area mesoporous silica matrix, containing commercial fumed silica as well as synthetic silica. The adsorbents exhibited a great CO_2_ adsorption capacity of up to 0.98 mmol/g in environmental conditions. Importantly, these materials showed great recyclability and regeneration capabilities. They could be fully regenerated and recycled at relatively low temperatures, as low as 348 K, with more than 85% retention of their adsorption capacity after four cycles. This characteristic makes them highly valuable materials for CCS technologies [99].

Song et al. utilized a low-cost quaternized chitosan/PVA hybrid aerogel to adsorb CO_2_ reversibly from ambient air. The determined CO_2_ capture capacity of the aerogels was approximately 0.18 mmol/g [100].

In another study, a sustainable and environmentally friendly approach was tested for the production of chitosan-grafted graphene oxide aerogels as adsorbents for CO_2_. Chitosan was cross-linked with graphene oxide and multi-walled carbon nanotubes to create adsorbents with large surface areas, high porosity, and numerous amine groups, which contribute to CO_2_ adsorption. The adsorption capacity of CO_2_ was approximately 0.257 mmol/g at 1 bar. This capacity was drastically greater compared to the adsorption capacity of chitosan alone [101]. 

Primo et al. showed the synthesis of carbon spheres from alginate and chitosan, by thermal treatment (400 < *t* < 1273 K, inert atmosphere). Both the raw natural biopolymers and the subsequent carbon materials presented a significant CO_2_ adsorption capacity of 5 mmol/g measured at 273 K and atmospheric pressure [102]. This adsorption capacity is similar to the given record for CO_2_ adsorption and significantly higher when compared based on unit areas or material density. The elevated nitrogen content already contained in the biopolymer and its high microporosity are essential issues that contribute to achieving such satisfactory adsorption values with a simple and environmentally friendly preparation procedure. Table 4 shows a summary of the biopolymer-based materials used for CO_2_ capture, with the related capture methods and absorption/adsorption capacity.

**Figure 8:**
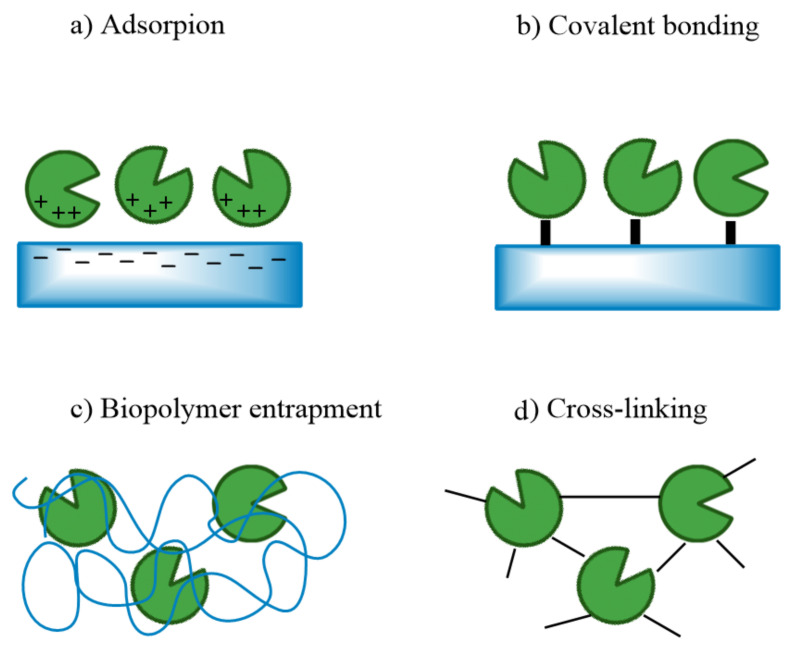
Immobilization techniques: (**a**) adsorption, (**b**) surface covalent bonding, (**c**) encapsulation within a polymer, and (**d**) cross-linking.

Reviewing the recent and less recent scientific literature, it is possible to find works where alginate and chitosan are combined to induce a biomimetic carbon capture process through the immobilization of an enzyme, carbonic anhydrase (CA), through which CO_2_ is absorbed followed by the production of carbonates [103]. The immobilization also enables facile separation of the enzyme from the reaction solution, simplifying downstream processing. However, a major challenge in this process is to find an efficient immobilization method for enzyme reuse. Different immobilization techniques are possible, such as covalent bonding, cross-linked enzyme aggregates, adsorption, entrapment, or a combination of the above (Figure 8). Both alginate and chitosan provide a favorable microenvironment for the encapsulation of CA, protecting it from denaturation and enhancing its stability. 

Table 5 reports the most recent research on CO_2_ captured by CA immobilized in alginate/chitosan composite materials, highlighting activity, thermal and storage stability, and CA reusability, whereas Table 6 lists the main advantages and disadvantages of using sodium alginate and/or chitosan nanocomposites.

The chitosan- and alginate-based materials described above have excellent properties that make them particularly suitable for the reversible adsorption of CO_2_. These properties are implemented through appropriate chemical modifications and include an adequate surface area, the presence of N atoms (naturally present in chitosan), high density, and electrical conductivity. An industrial scale-up of the use of chitosan/alginate nanocomposite materials in CO_2_ capture can be feasibly anticipated since both biopolymers are already employed in large-scale production processes. 

### 3.3. Carrageenan

**Figure 9:**
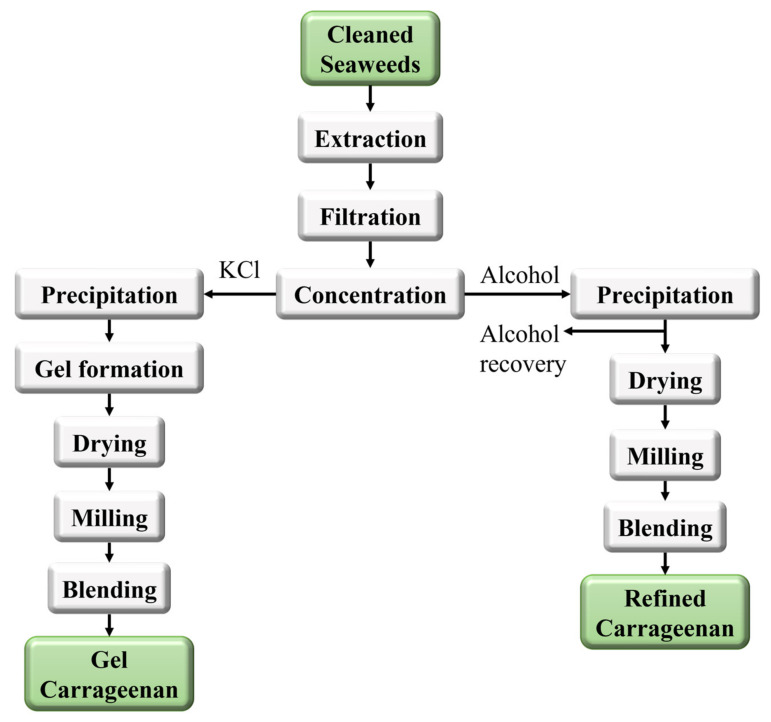
Flowchart for the extraction of gel and refined carrageenan from seaweeds [110].

Carrageenan is a natural polysaccharide extracted from red seaweed characterized by sulfated D-galactopyranose units, variably linked in a linear polymer [8]. It has captured interest in various fields, including food, pharmaceuticals, and biomedical applications. The exhaustive carrageenan extraction methods are usually trade secrets of the manufacturers; however, on a larger scale, they usually follow a similar pattern to that shown in Figure 9, which describes the main steps involved in the production of carrageenan. 

In recent years, researchers have also explored its potential use in environmental applications, such as CO_2_ capture, presenting several advantages with respect to the capture from systems directly based on microalgae. These latter types have been extensively studied, and it has been experimentally shown that the introduction of fumes and exhaust gases into these systems damages chloroplasts, interrupting photosynthesis and other metabolic processes in the microorganisms, thus limiting the absorption of CO_2_. In addition, the process of immobilizing microalgae involves various efforts, with the need to control various environmental parameters such as illumination and pH. In particular, κ-carrageenan was employed as a biopolymer to entrap and protect microalgal cells from environmental stresses and to form a novel trickle bed reactor with immobilized algae to adsorb CO_2_ from the exhaust and flue gas [111].

A recent study reported the synthesis and characterization of S and N doubly-doped high-surface-area κ-carrageenan. The biopolymer was modified by adding graphene oxide (GO) or carbon nanotubes (CNTs), resulting in changes to the porous structure and surface chemistry of the material [112,113]. The *S*- and *N*-doped κ-carrageenan functionalized with GO and CNTs had significantly higher surface areas as compared to the nanoparticle-free biopolymeric matrix (κ-carrageenan: 1070 m^2^/g; +GO: 1780 m^2^/g: +CNTs: 1170 m^2^/g). This indicates that the addition of carbon nanocharges improved the surface area and increased the material’s porosity, which could have beneficial outcomes in gas adsorption and separation processes [113].

Epuran et al. developed a multifunctional carboxyl-substituted porphyrin/κ-carrageenan composite capable of both detecting/capturing carbon dioxide and monitoring toxic metal ions such as Mn^+2^ from waters. Porphyrin dyes are very well known for their sensing capabilities thanks to very strong absorption, with extinction coefficients around 10^5^ M^−1^ cm^−1^, and an ability to sense the pH, the presence of metal ions in the environment, as well as to report information on the local molecular environment [114,115]. Thus, when combining the biopolymer with the dye, this leads to composites able to contribute to environmental monitoring while maintaining a sustainable environment. Overall, 1 g of this porphyrin/κ-carrageenan nanocomposite with 0.09 g porphyrin can adsorb 6.97 mmol of CO_2_ in ambient conditions [116]. This outcome is six times worse than the best demonstrated one, i.e., of 42 mmol/L for each g of adsorbent material, a result, however, that was obtained under high temperature (373 K) and pressure (10 bar) [116]. 

In another investigation, a new biocomposite was produced by anchoring living microalgae, specifically *Chlorella vulgaris*, to textiles, repurposing them to serve as a platform for CO_2_ capture. This result was obtained by coating cotton or polyester with a κ–carrageenan gel and using these as a solid substrate for affixing *Chlorella vulgaris* and enhancing microalgae retention [117]. The cotton-based biocomposites exhibited notably higher CO_2_ absorption than suspended microalgae cultures, achieving a peak CO_2_ absorption rate of 1.82 g of CO_2_ per gram of biomass per day from the coated biocomposites. However, the CO_2_ absorption rates of coated and uncoated polyester biocomposites were comparatively lower (0.49 and 0.42 g CO_2_ per gram of biomass, respectively), probably due to surface charges impacting microalgae adhesion and retention [118]. After assessing the microalgae attachment on cotton/polyester blends over two weeks, some degradation was observed in the textile, potentially limiting the durability of the biocomposites [119].

Some recent studies on CO_2_ capture by κ-carrageenan biocomposite materials are summarized in Table 7, while Table 8 lists the main advantages and disadvantages of using carrageenan in a composite nanomaterial.

## 4. Industrial Scale-Up

The use of biopolymeric composite materials in carbon dioxide capture presents several industrial aspects, which include both opportunities and challenges [86]. Numerous obstacles hinder the widespread adoption of bio-based sorbents. One such challenge lies in the degradation or decomposition of these adsorbents at elevated temperatures, a significant concern within this domain [120]. Also, given the nature of biomaterial-based sorbents, the synthesis of highly uniform and chemically homogeneous materials poses a critical challenge. Hence, ensuring quality control at various stages of the process is strongly recommended to maintain consistent separation performance throughout carbon capture and storage operations. As bio-based sorbents exhibit a limited adsorption capacity compared to other sorbents, their replacement or reactivation becomes necessary upon observing a decline in efficiency [121,122]. Additionally, the regeneration process hinges on the adsorption mechanism and mechanical stability of the material. Thus, careful selection of the process configuration, including the regeneration pathway, becomes imperative not only from a cost perspective but also considering potential impacts on sorbent stability and surface functional groups. Moreover, the efficacy of applied chemical treatments may vary and could potentially be detrimental, depending on the physicochemical properties of the initial biomass. Adsorption technologies, akin to membrane separation ones, are better suited for low recovery rates and small-scale operations, whereas absorption-based capture methods boast a broader applicability range [123]. Bridging the gap between commercialized amine solutions and bio-based sorbents remains a challenge, with moisture resistance being a key issue for the latter, often necessitating the pre-drying of flue gas [124]. Potential sorbent development efforts should prioritize aspects such as recovery, reusability, and humidity resistance to enhance competitiveness in the market. Additionally, improving the cyclability of bio-based sorbents is a crucial advancement. For example, various bio-based sorbents derived from different precursors exhibit varied CO_2_ adsorption capacities across multiple cycles. Despite some outliers demonstrating efficient CO_2_ adsorption capacity even after ten cycles, challenges persist in biomass-based carbon aerogel synthesis, primarily related to the time-consuming and expensive synthesis process and limitations in microstructure modification. Further research is needed to optimize operational procedures, prevent micropore structure breakdown, and enhance membrane selectivity. Addressing operational challenges related to improved sorbent nano/microparticle shift at elevated gas flow rates requires exploring immobilized particle forms for improved applicability and facilitated recovery. Additionally, the life cycle of bio-based sorbents warrants equal consideration, with life-cycle assessment (LCA) emerging as a crucial tool for estimating environmental impacts throughout the sorbent’s entire life cycle [125]. Strategies such as reusing spent bio-based sorbents as biofuel or catalysts for multiple applications, and employing renewable energy sources are highly sought after to enhance the efficiency and sustainability of carbon capture processes [121]. Conducting LCA and whole system analyses can further validate the value of emerging technologies in this field, providing insights into energy consumption, environmental impacts, and overall system efficiency.

## 5. Conclusions and Future Perspectives

As more and more evidence is collected, the role of carbon dioxide (CO_2_) as a greenhouse gas and a significant contributor to global warming cannot be underestimated. The heightened greenhouse effect due to increased CO_2_ levels has instigated widespread climate change concerns, necessitating proactive measures. CO_2_ capture emerges as a vital strategy to curb the release of carbon dioxide emissions from industrial and power generation processes into the atmosphere. The primary objective of this approach is to mitigate the adverse anthropogenic effects on the Earth’s climate.

One promising avenue in the field of CO_2_ capture involves the use of biopolymer nanocomposites. These innovative materials leverage the renewable and sustainable attributes of biopolymers, which are derived from natural sources, combined with nanofillers that enhance the overall performance of the composite. Diverse biopolymers, including chitosan, cellulose, carrageenan, starch, alginate, and various proteins, exhibit unique functional groups that facilitate interactions with CO_2_ molecules.

The incorporation of nanofillers into biopolymer matrices has shown significant potential in enhancing mechanical, thermal, and sorption properties. Materials like graphene, carbon nanotubes, metallic nanoparticles, MOFs, and zeolites have demonstrated a capacity to elevate the surface area and porosity of biopolymer nanocomposites, leading to improved CO_2_ adsorption capabilities.

The CO_2_ capture mechanism within biopolymer nanocomposites revolves around chemisorption and physisorption. The functional groups inherent in the biopolymer matrix, such as amino and hydroxyl groups, enable weak interactions that contribute to CO_2_ adsorption. The introduction of nanofillers further amplifies the adsorption capacity by augmenting surface area and porosity.

Numerous advanced materials, including biopolymer derivatives, have been developed for CO_2_ capture technology, incorporating absorption, adsorption, and membrane separation techniques. Biopolymers like cellulose, alginate, chitosan, and carrageenan, along with their derivatives, offer accessibility and cost-effectiveness.

This review addresses recent studies on the strategies for CO_2_ capture involving biopolymer-based materials. It provides an encompassing overview of composite materials that merge cellulose, alginate, chitosan, and carrageenan biopolymers with nanomaterials, highlighting their potential. By surveying the literature on these specific biopolymers, we hope to bring further critical insight into their use in reducing environmental CO_2_, alongside numerous review studies on their applications in many other technologically relevant fields. This review delves into the synthesis and efficiency of these materials, hinting at a trend towards enhanced CO_2_ capture technology on a global scale. Among the materials scrutinized, an amino-functionalized alginate-based adsorbent exhibited the highest adsorption capacity, outperforming other options, followed by a novel approach utilizing κ-carrageenan-based biocomposites integrated with living algae as solid substrates for CO_2_ capture.

In summary, the integration of biopolymer nanocomposites for CO_2_ capture holds considerable promise in the quest to mitigate climate change. As research and technology continue to advance, these innovative materials may pave the way for more effective and sustainable solutions to the ongoing challenge in reducing carbon emissions and in mitigating their impact on our environment. However, significant research, development, and innovation efforts are required to overcome technical challenges and realize the full potential of these materials in industrial applications.

## Figures and Tables

**Table 1:** Chemically modified cellulosic materials for CO_2_ adsorption.

Cellulose Derivative	Chemical Modification	CO_2_ Adsorption Capacity (mmol/g)	Notes	Refs.
CNFs aerogel	3-Aminopropylmethyl-diethoxysilane	2.26	Absorption capacity increases linearly with humidity	[51]
CNFs foam	PEI ^a^	2.22	Reduced surface area after modification	[52]
CNC composite	Silica, triethoxysilylpropyl-3-pentanyldinitrile carbamate	5.54		[53]
CNFs aerogel	*N*-(2-aminoethyl)-3-aminopropylmethyldimethoxysilane and acetic acid	1.91	Reduced surface area after modification	[54]
CNFs aerogel	*N*-(2-aminoethyl)-3-aminopropylmethyldimethoxysilane	1.78	Reduced surface area after modification	[55]
CNFs aerogel	Silica, Na_2_SiO_3_, APTES ^b^	2.2	Improved surface area with silica incorporation; reduced surface area after silanization.	[56]
CNFs thin film	(3-trimethoxysilylpropyl) diethylenetriamine	2.11		[57]
CNC aerogel	*N*-(2-aminoethyl)-3-aminopropylmethyldimethoxysilane	1.68	Reduced surface area after modification	[58]
CNFs foam	Silicalite-1 zeolite	1.2		[59]
CNFs foam	ZIM ^c^	0.62		[60]
Cellulose	(3-Chloro-2 hydroxypropyl) trimethylammonium chloride	0.14		[61]
CNFs aerogel	*N*-(2-aminoethyl)-3-aminopropylmethyldimethoxysilane	1.01	Reduced surface area after modification	[5]
CNFs aerogel	*N*-(2-aminoethyl)-3-aminopropylmethyldimethoxysilane	1.59	Reduced surface area after modification	[62]
CNFs aerogel	phthalimide (1,3-dihydro-1,3-dioxoisoindole)	5.3		[63]
CNFs aerogel	Sodium acetate	1.14		[64]
Cellulose aerogel	Acrylamide	1.07		[65]
Cellulose aerogel	Silica	1.96–11.87	Gas selectivity increases with silica gel content.	[66]

^a^ Polyethylenimine; ^b^ 3-aminopropyl triethoxysilane; ^c^ zeolitic imidazolate framework.

**Table 2:** Chemically modified cellulosic membranes for CO_2_ adsorption.

Cellulose Membrane	Chemical Modification	CO_2_ Permeability/Permanence	CO_2_ Selectivity	Refs.
Polysulfone	CNF/polyvinyl amine coating	25 Barrer	CO_2_/N_2_: 500 CO_2_/CH_4_: 350	[69]
Regenerated cellulose	-	155.0 Barrer	CO_2_/N_2_: 27.2	[70]
Cellulose acetate	Amine functionalized MIL-53(Al) ^a^	52.6 Barrer	CO_2_/N_2_: 23.4	[71]
Cellulose acetate	Poly(ionic liquid)	8.9 Barrer	CO_2_/N_2_: 26.8	[72]
Polysulfone	PVA/CNC ^b^ coating	0.27 m^3^(STP)/(m^2^⋅bar⋅h)	CO_2_/CH_4_: 39	[73]
Polyvinylamine	CNF	187 Barrer	CO_2_/N_2_: 100	[74]
CNF	UiO-66 ^c^	139 Barrer	CO_2_/N_2_: 43.6	[75]
Ethylcellulose	ZIF-8 ^d^ ZIF-8/graphene oxide	203.3 Barrer	CO_2_/N_2_: 33.4	[76]
Cellulose acetate	Vinyltrimethoxysilane with acetic acid	24.5 Barrer	CO_2_/CH_4_: 28.8	[77]
Cellulose diacetate	–	9 Barrer	CO_2_/CH_4_: 30–35	[78]
Polysulfone	PVA/phosphoryl-CNC coating	0.21 m^3^(STP)/(m^2^⋅bar⋅h)	CO_2_/CH_4_: 46	[79]
Polysulfone	PVA/CNC Phosphorylated CNF Oxidized CNF coating	27.8 ± 5.5 GPU ^e^; 100 ± 3.7 GPU; 90.7 ± 3.7 GPU	CO_2_/N_2_: 39 ± 0.4; 42 ± 1.8; 90.7 ± 3.7; 42 ± 0.7	[80]
PVDF ^f^	PVA/polyallylamine/functionalized CNF coating	652 GPU	CO_2_/N_2_: 41.3	[81]
PPO ^g^	PVA/CNC coating	672 GPU	CO_2_/N_2_: 43.6	[82]
Regenerated cellulose	PEI-modified graphene oxide	268.9 Barrer	CO_2_/N_2_: 48.9 CO_2_/CH_4_: 57.4	[83]
Cellulose triacetate	–	110 GPU	CO_2_/CH_4_: 22	[84]
CNF	ZIF-8	550 Barrer	CO_2_/N_2_: 45.5 CO_2_/CH_4_: 36.2	[85]

^a^ XIII–benzene-1,4-dicarboxylate, (X = Al, Fe, Ga, Cr, Sc, In); ^b^ Poly(vinyl alcohol); ^c^ Zr-based MOF; ^d^ Zeolitic imidazole framework; ^e^ Gas permanence unit; ^f^ Polyvinylidene fluoride; ^g^ Poly(p-phenylene oxide).

**Table 3:** Advantages and disadvantages of cellulose composite nanomaterials.

Advantages	Disadvantages	Refs.
Abundant and renewable resource, primarily derived from plant sources	Poor solubility in most common solvents, requiring specialized processing methods	[63]
Biodegradable and environmentally friendly	Limited thermal stability	[74]
High strength and stiffness, making it suitable for reinforcing composite materials	Susceptible to degradation by microbial activity under certain conditions	[81,83]
Good compatibility with other materials due to its hydrophilic nature	Processing can be energy-intensive and require expensive processes	[61]
Can be easily processed into 0D to 2D nanostructured materials (nanoparticles, fibers, films)		[78,80,81]

**Table 4:** Chemically modified alginate or chitosan materials for CO_2_ capture.

Biopolymer	Chemical Modification	Mechanism of CO_2_ Capture	CO_2_ Captured (mmol g^−1^)	Refs.
Alginate	PVA ^a^, ZIF ^b^	Membrane gas separation	-	[96]
	NH_2_-functionalized	Adsorption	0.2380 ^c^	[97]
	NH_2_-SiO_2_	Adsorption	7.865 ^d^	[88]
	CA ^e^	Absorption	0.025 ^f^	[98]
Chitosan	SiO_2_	Adsorption	0.98	[99]
	PVA	Adsorption	0.18	[100]
	GO ^g^ or MWCN ^h^	Adsorption	0.257	[101]
Alginate and Chitosan	Pyrolyzed	Adsorption	5	[102]

^a^ Poly(vinyl alcohol); ^b^ Zeolitic imidazole framework; ^c^ CO_2_ 45%, *p* = 1 bar *t* = 303 K; ^d^ CO_2_ 5%, gas flow rate of 40 mL/min *t* = 313 K; ^e^ Carbonic Anhydrase; ^f^ 1 mg of enzyme; ^g^ Graphene Oxide; ^h^ Multi-walled carbon nanotube.

**Table 5:** CA immobilization in alginate/chitosan biopolymer and related enzymatic activity, stability, storage, and reusability.

CA Source	CA Immobilization Technique	Activity ^a^	Thermal Stability ^b^/Storage ^c^	CA Reusability ^d^	Refs.
Bovine	Entrapment	30.8	~35.7/7.1 (3 h, 343 K)	-	[104]
Purified bacterial		94.5	43.3 (2 h, 343 K)/81.2 (28 d, 277 K)	53% (8 c)	[105]
Mammals/extremophile bacteria		60	-	-	[103]

^a^ % k_cat_/K_m_ or V_max_/K_m_ immobilized to free CA ratio; ^b^ maximum immobilized CA activity/relative free CA activity in % (incubation time, temperature); ^c^ initial immobilized CA activity/relative free CA activity in % (storage time, temperature); ^d^ first-cycle immobilized CA activity.

**Table 6:** Advantages and disadvantages of sodium alginate and/or chitosan composite nanomaterials.

Biopolymer	Advantages	Disadvantages	Refs.
Sodium alginate	Derived from seaweed and algae, thus a sustainable and abundant resource	Limited mechanical strength compared to synthetic polymers	[102]
	Biocompatible and non-toxic	Susceptible to enzymatic degradation in the presence of alginate lyases	[102]
	Forms hydrogels with divalent cations, offering versatility in material properties	Solubility and gelation properties can be affected by pH and temperature	[96]
	Good film-forming ability, enabling the production of thin films for various applications	Relatively high cost compared to some other natural polymers	[106]
	Can be easily cross-linked to improve mechanical properties and stability		[106]
Chitosan	Derived from chitin, a biopolymer found in fungi and arthropods	Limited solubility in water at neutral pH, requiring acidic conditions for dissolution	[102]
	Biocompatible, biodegradable, and non-toxic	Mechanical properties possibly affected by moisture absorption	[99]
	Antimicrobial properties	Challenging solution processing due to high viscosity	[92]
	Forms films, gels, and fibers with excellent mechanical properties	Sensitive to enzymatic degradation by chitinases	[107,108,109]
	Can be chemically tailored to introduce specific functionalities		[107,109]

**Table 7:** Chemically modified κ-carrageenan biocomposite materials for CO_2_ adsorption.

κ-Carrageenan Biocomposite	CO_2_ mmol g^−1^ Adsorbent	Refs.
*Chlorella vulgaris* on cotton sheet	41.29 ± 2.17	[119]
*Chlorella vulgaris* on polyester sheet	11.09 ± 0.85	[119]
Carboxyl-substituted porphyrin	6.97	[116]

**Table 8:** Advantages and disadvantages of carrageenan composite nanomaterials.

Advantages	Disadvantages	Refs.
Extracted from red seaweed, making it a renewable and sustainable resource	Limited mechanical strength compared to synthetic polymers	[110]
Forms strong and flexible gels in the presence of potassium ions	Susceptible to degradation by microbial enzymes and acidic conditions	[116]
Excellent stabilizing and thickening properties in aqueous solutions	Gelation properties can be affected by the presence of certain ions and pH	[119]
Biocompatible and non-toxic		[110]
Can be modified to tailor its properties for specific applications		[113,116]
